# Faith after an Earthquake: A Longitudinal Study of Religion and Perceived Health before and after the 2011 Christchurch New Zealand Earthquake

**DOI:** 10.1371/journal.pone.0049648

**Published:** 2012-12-05

**Authors:** Chris G. Sibley, Joseph Bulbulia

**Affiliations:** 1 University of Auckland, Auckland, New Zealand; 2 University of New Zealand, Wellington, New Zealand; Universidad Carlos III de Madrid, Spain

## Abstract

On 22 February 2011, Christchurch New Zealand (population 367,700) experienced a devastating earthquake, causing extensive damage and killing one hundred and eighty-five people. The earthquake and aftershocks occurred between the 2009 and 2011 waves of a longitudinal probability sample conducted in New Zealand, enabling us to examine how a natural disaster of this magnitude affected deeply held commitments and global ratings of personal health, depending on earthquake exposure. We first investigated whether the earthquake-affected were more likely to believe in God. Consistent with the Religious Comfort Hypothesis, religious faith increased among the earthquake-affected, despite an overall decline in religious faith elsewhere. This result offers the first population-level demonstration that secular people turn to religion at times of natural crisis. We then examined whether religious affiliation was associated with differences in subjective ratings of personal health. We found no evidence for superior buffering from having religious faith. Among those affected by the earthquake, however, a loss of faith was associated with significant subjective health declines. Those who lost faith elsewhere in the country did not experience similar health declines. Our findings suggest that religious conversion after a natural disaster is unlikely to improve subjective well-being, yet upholding faith might be an important step on the road to recovery.

## Introduction

After the wind there was an earthquake, but the Lord was not in the earthquake. After the earthquake came a fire, but the Lord was not in the fire. And after the fire came a gentle whisper. Then a voice said to him, “What are you doing here, Elijah?”

**1 Kings 19: 11–13**

**Figure 1:**
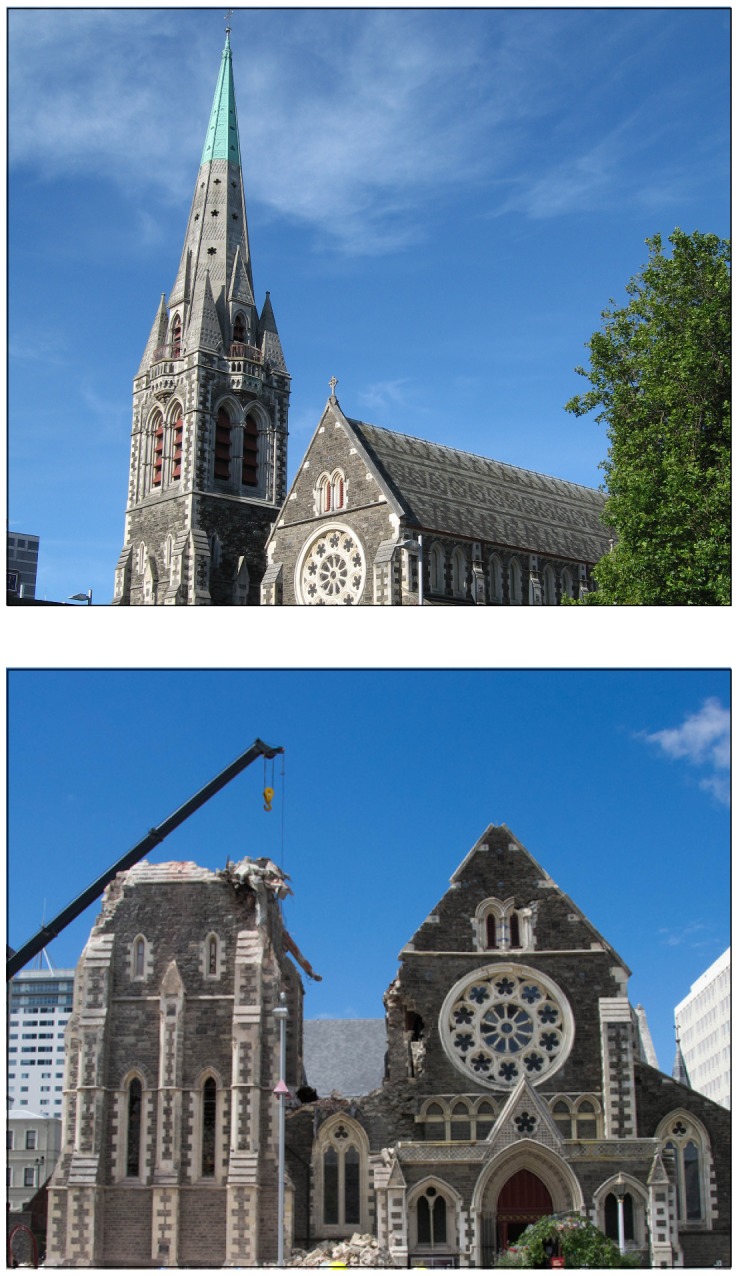
The top figure shows the Christchurch National Anglican Cathedral, a city landmark, before the 22 February 2011 earthquake. The bottom the figure shows the Cathedral after the earthquake. Images were taken from Wikipedia Commons subject to the GNU Free Documentation License.

At 12:51pm on 22 February 2011, a M_W_ 6.3 earthquake struck Christchurch New Zealand (population 367,700) at a depth of only five kilometres, resulting in New Zealand's most deadly natural disaster in eighty years. Two multi-story office buildings collapsed, igniting fires, killing one hundred and ten. Falling bricks and mortar crushed two city buses, killing six. Another sixteen died from falling debris and landslides. Many hundreds were injured. The Catholic Cathedral of the Blessed Sacrament was ruined. The cross atop its dome was set to lean. The Anglican Cathedral at Christchurch was shattered. Its brick spire, a city landmark, was toppled (Figure 1.) Emergency services were overwhelmed. Hospitals were flooded. Power and water were lost. Phone lines were downed. Many who worked in the city were isolated from their families. After surveying the damage, Prime Minister John Key told the media: “It is just a scene of utter devastation. We have to work as fast as we can to get people out of environments where they are trapped. This is a community that is absolutely in agony” (source BBC News, website accessed: http://www.bbc.co.uk/news/world-asia-pacific-12533291. Accessed 17 October, 2012.) This agony was anticipated by a less deadly but larger earthquake on 4 September, 2010 (M_W_7.1), and aftershocks rattled Christchurch for many months after the February event. All told, roughly a third of the buildings in Christchurch were damaged beyond repair (source: New Zealand Treasury, website accessed 17 2012 October: http://www.teara.govt.nz/en/historic-earthquakes/13). The New Zealand Treasury estimated the total costs for rebuilding Christchurch to be NZ$ 20 billion, or about US$ 15 billion (source New Zealand Treasury, website accessed 17 October 2012: http://www.treasury.govt.nz/budget/2012/speech/07.htm). Residents were left in doubt for months about the extent of government and private insurance, prolonging their agony.

The earthquakes and their aftermath occurred between the 2009 and 2011 waves of the New Zealand Attitudes and Values Study, a large, countrywide longitudinal survey assessing change and stability in values (see NZAVS website, accessed 17 October 2012: http://www.psych.auckland.ac.nz/uoa/NZAVS). The timing of the earthquakes between two survey points afforded the unprecedented opportunity to examine how a natural disaster of this magnitude affects deeply held religious commitments and global ratings of personal health.

We were interested in two questions. It has long been argued that people turn to religion during times of crisis [1], [2]. Our first set of analyses investigated whether the earthquake affected religious conversion rates (religious affiliation). It has also been argued that religious affiliation benefits the faithful at times of extraordinary grief, anxiety, and danger [3], [4]. Our second set of analyses investigated whether religious affiliation was associated with overall higher subjective health ratings for those who were affected by the earthquake relative to those who were not.

### Theories of religious comfort

Philosophers have long maintained that religion functions as an emotional firebreak against anxiety [5] and suffering [2], and is especially appealing after natural calamities [1]. Sigmund Freud characterises the problems to which religion offers consolation as follows:

No one is under the illusion that nature has already been vanquished there are elements, which seem to mock at all human control: the earth, which quakes and is torn apart and buries all human life and its works; water, which deluges and drowns everything in a turmoil; storms, which blow everything before them; there are diseases, which we have only recently recognised as attacks by other organism, and finally there is the painful riddle of death, against which no medicine has yet been found, nor probably will be. With these forces nature rises up against us, majestic, cruel and inexorable; she brings to our mind once more our weakness and helplessness *Future of an Illusion* (pp.15–16).

Recent laboratory studies offer preliminary support for the Religious Comfort Model. Researchers have discovered that religious faith becomes more appealing after reminders of tragic suffering [6], danger [7], randomness [8] and death [9]. Other studies show that experiences of suffering tend to intensify religious sentiments, an effect that is well explained by comfort-seeking [10]. The appeal of religion during crisis has been observed to affect large populations. In a study on religious responses to the September 11, 2001 attacks on the United States, Trevino and Pargament found that seventy-five percent of a national sample reported moderate or heavy reliance on prayer to cope with their stress [11].

To our knowledge, no previous study has investigated whether the appeal of religion following a large-scale disaster is sufficient to affect rates of religious conversion. The timing of the Christchurch earthquakes between two NZAVS survey points enabled us to test the predictions of the Religious Comfort Model by comparing conversion rates in populations who were personally affected by the earthquake with populations who were not.

#### The Religious Comfort Hypothesis

those affected by the Christchurch earthquakes or living in the region of the earthquakes will be more likely to retain/convert to religion when compared with those who were not affected by the earthquakes or who were living in different regions.

We emphasise that the Religious Comfort Hypothesis predicts that higher conversion rates will only occur among those affected by the Christchurch earthquakes. Comparisons with the rest of the New Zealand enabled us to better understand the specific contribution that the earthquake and its aftermath made to conversion rates.

### Theories of religious buffering

#### Superior religious buffering

It has been proposed that in the wake of unusually stressful events, religious comforts offer practical benefits, however ideas about such benefits vary. Suggestions include that religious belief: (1) supports placebo healing [12]; (2) bolsters cooperation and solidarity [13]–[18]; and (3) brings healthful reductions to anxiety [4], [19]. A tidal surge of research in the evolutionary study of religion has focussed on how such benefits explain the remarkable evolutionary endurance of religion [20]–[38].

Notably, many researchers remain skeptical about religious buffering. Karl Marx famously argued that religion persists as an empty distraction from the conditions of suffering [2]. More recently, it has been argued that religion is best approached as culturally transmitted error, a kind of virus of the mind [39], which may intensify certain forms of suffering [40]. Some researchers urge that religious practices suppress anxieties only temporarily, perpetuating a long term demand for religion [41].

Against dysfunctional theories, numerous studies have found that religion improves health, facilitates coping, [42], [43] and improves social belonging [44]. Moreover religion has been shown to improve emotional regulation [45] and is associated with diminished conflict monitoring for stressful events [46]. For example, religious parents appear to be better able to cope with the loss of a child [47]. Related studies have found that religion spikes early during bereavement, and those who turn to religion more experience less grief later on [48]. In a landmark study, Sosis and Handwerker (2011) compared the effects of Psalm recitation for women close to the lines of 2006 Israel/Lebanon War with women who had migrated to safety [49]. The investigators found that Psalm recitation was associated with lower rates of anxiety in the warzone but not elsewhere. This result supports Bronislaw Malinowski's theory that supernatural thinking (“magic”) arises “wherever the elements of chance and accident, and the emotional play between hope and fear have a wide and extensive range. We do not find magic wherever the pursuit is certain, reliable, and well under the control of rational methods and technological processes” (p.116) [50].

It is important to note that the role religious faith plays in facilitating coping is often difficult to disentangle from other factors, such as community help and norms for healthy lifestyles. Religious denominations also vary in the support they provide for different stressors – Catholics, for example, find it harder to overcome divorce [51]. Further variability has been found for the effects of religious practice on different personality types – those high in neuroticism feel more social belonging from religious attendance [44]. Complex and interrelated dimensions of variability complicate simple stories about how religion buffers people from anxiety and grief. Yet if Malinowski's model were to generalise to New Zealand, we might expect that religious faith in the aftermath of the Christchurch earthquake – a devastating, uncontrollable, and unpredictable natural disaster – would tend to be associated with superior buffering effects for subjective well-being.

#### The Superior Religious Buffering Hypothesis

religious believers affected by the Christchurch earthquakes or living in the region will report superior health when compared with disbelievers.

Here again, we used response data from those living outside of Christchurch who were not affected by the earthquakes to identify the specific effects of the natural disaster and aftermath, as compared to general processes that affected everyone in New Zealand, on subjective health.

#### Selective religious buffering

By world standards, New Zealand is a highly secular country, and it has grown increasingly secular during the past half century [52], [53]. We might therefore expect that secular institutions have evolved to perform functionally equivalent roles to religion [54], [55]. Experimental and correlational evidence indicating functional similarities in religious and secular buffers comes from a study by Kay and colleagues who found that confidence for religion and government exhibit hydraulic dependencies, such that lowering confidence for one source of control increases confidence for the other [56]. To the extent that such processes are reflected in behaviours, we might expect that religious and secular people would derive comparable advantages from their orientations. Further evidence for this prospect comes from W.L. Frazier's Ph.D. dissertation, which investigated whether religion improved mental health following 2005 Hurricane Katrina, the third most deadly hurricane in U.S. history [57]. Frazier found that the effects of religious forms of coping were not distinguishable in their effects from general forms of non-religious coping strategies. Many social researchers suggest that traditional religious faith might be declining in affluent democracies because secular institutions have emerged as surrogates for religion, offering existential security [58], [59], assuring income [60], and furnishing superordinate identities and sentiments [61], replacing the functions that traditional religions performed in the past [62].

Though the overall benefits of religious and secular buffers might be similar, access to secular benefits might plausibly differ. At a philosophical level, religious orientations appear to provide all-embracing world-views with highly prescribed norms and practices [26], [63]. By contrast, secular philosophies appear to be relatively diffuse, unstructured, and variable [64], and secular philosophies accord comparatively wide freedoms to individuals for deciding matters of right and wrong [65]. At a community level, religious groups have been found to provide exceptionally strong levels of emotional support [44], [66]. During crisis, religious community support may extend to financial insurance [67]. By contrast, secular groups are less readily identifiable as communities of moral concern [53], [68]. In New Zealand, moreover, insurance is largely left to market financial sectors. Knowing nothing else, it would seem plausible that accessing the benefits of religious theologies/networks in the wake of a terrible natural disaster will occur relatively quickly for religious converts, yet accessing the benefits of secular philosophies/networks will be more difficult for those who lose their religion. At times of extraordinary stress, we might expect this differential access to bring additional health risks, in which case religion would offer selective buffering effects. Notably, the media have criticised insurance companies for inadequate coverage, poor communication, and slow response; the most obvious secular buffering institutions have been judged to have performed poorly [see for example **The Listener**, accessed on 18 October 2012: http://www.listener.co.nz/current-affairs/christchurch-homeowners-face-long-wait-for-rebuild/].

Preliminary evidence for selective buffering comes from a recent study by Seirmarco and colleagues, who drew on retrospective reports about the importance of religion three years after the September 11 attacks [69]. The investigators found that decreases in the recollected importance of religious belief after the attacks (ten percent of the sample) were strongly correlated with poorer mental health outcomes. On the other hand, those who recollected increased importance of religious belief (eleven percent of the sample) did show improved mental health when compared with those who reported no change in their religion. This correlational finding suggests that the loss of religion might be selectively damaging to personal well-being. However the authors point out that stated declines in the importance of religion were associated with the reported severity of personal loss during the attacks. This finding complicates a selective buffering interpretation because the loss of faith might have been provoked by exceptional grief. Lacking historical and comparative data it is difficult to evaluate the processes by which this tragedy affected health outcomes. Bering these limitations in mine, Seirmarco et al. 's study is important because it points to the possibility that the loss of religion might be associated with declines in psychological well-being, even if maintaining or acquiring religion is not associated with improvements. If secular buffers were harder to access, we would expect that those who lost their religious faith and were affected by the Christchurch earthquakes would exhibit poorer subjective health, from a combination of earthquake hardships, loss of religious buffering, and the inability to readily fill that void.

#### The Selective Religious Buffering Hypothesis

those affected by the Christchurch earthquakes who lose religious faith (apostates) will experience health declines relative to believers and longer-standing secularists.

The distinction between superior and selectively buffering requires a corresponding distinction between four categories of religious orientation:

**Believers**: those who identified as religious both in 2009 and in 2011.

**Converts**: those who were not-religious in 2009 and changed to a religious identification in 2011.

**Disbelievers**: those who were neither religious in 2009 nor in 2011.

**Apostates**: those who were religious in 2009 and changed to a not-religious identification in 2011.

We compared changes in ratings of subject health for participants from each of these four categories by examining differences both across regions, and based on participant self-reports of being personally affected or unaffected by the earthquakes. These methods enabled us to distinguish earthquake-related interactions of subjective health and religious affiliation from general processes affecting the entire population.

## Results

### Effects of the earthquake on conversion rates

#### Countrywide change in religiosity

Proportional rates of change and stability in religious affiliation in the New Zealand population from 2009 to 2011 are presented in Table 1.As reported in the table, countrywide comparisons of apostasy during the two-year interval across New Zealand showed a de-conversion rate of 6.6%, slightly higher than the proportion of religious conversions across New Zealand at 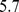
%. The net reduction in overall religious affiliation among the general population of New Zealand was 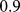
%.

**Table 1:** Proportion of sample that maintained or changed their religious affiliation from 2009 to 2011.

Subcategory	Result(%)
Believers	1460 (39.0)
Converts	214 (5.7)
Apostates	248 (6.6)
Disbelievers	1823 (48.7)

#### Change in religious belief depending on earthquake exposure

**Figure 2:**
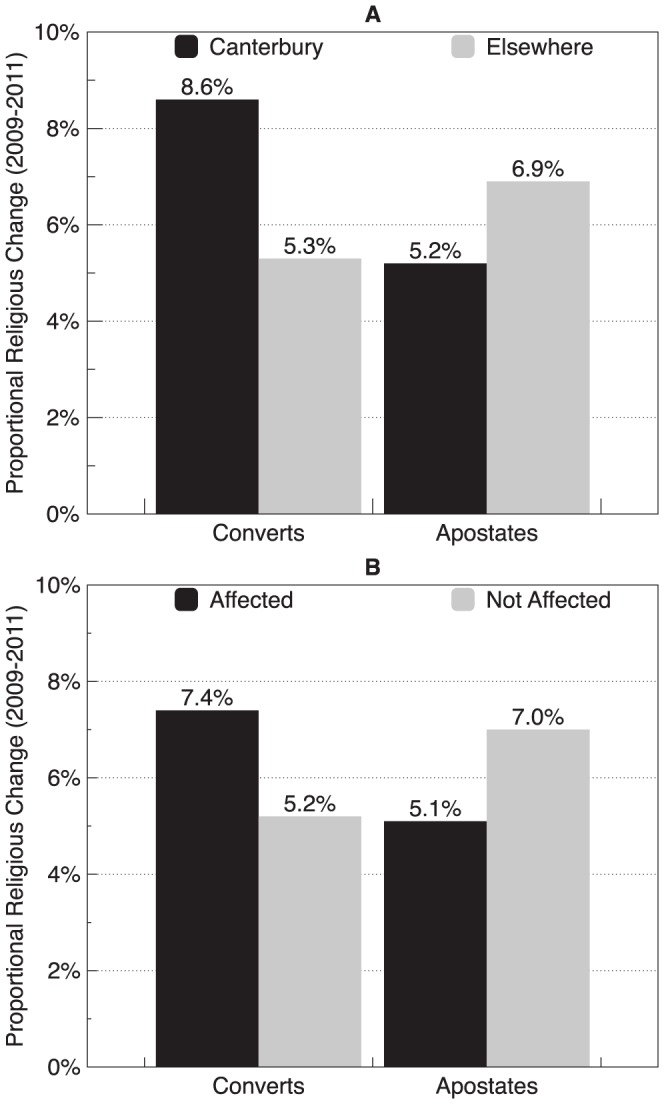
Bar graphs showing proportional rates of growth and decline in religious faith in the New Zealand population from 2009 to 2011. [**A**] describes religious change among those who lived in Canterbury (the region of the earthquakes). [**B**] describes religious change among those who reported being affected by the earthquakes and those who reported not being affected.

To determine rates of religious change among the earthquake-affected, we conducted parallel analyses using two measures of earthquake exposure: (a) by identifying participants by region of the earthquake (Canterbury) (b) by asking participants in the 2011 questionnaire to report whether they had been personally affected by the Christchurch earthquakes. Comparisons using regional data to examine change in religiosity among the New Zealand population affected by the Christchurch earthquakes are presented in Table 2. Comparisons using the report-of-being-personally-affected-by-the-earthquake data to examine change in religiosity among the New Zealand population affected by the Christchurch earthquakes are presented in Table 3. The proportion of believers and converts who experienced the Christchurch earthquakes as compared with those who did not are presented in Figure 2., which aggregates the raw data from Table 2. and Table 3. As shown in Figure 2., there was an overall countrywide decline in religious faith of roughly 0.9% each year.This rate of decline matches what Hoverd (2008) found in his historical analysis of New Zealand's religious trends, which showed a gradual erosion of religious faith among New Zealanders over the past 40 years [52] .

**Table 2:** Proportion of sample that maintained or changed their religious affiliation from 2009 to 2011 across the Canterbury region and other regions of New Zealand.

Category	Result(%)	Result(%)
	Canterbury region	Other regions
Believers	178 (35.5)	1281 (39.5)
Converts	43 (8.6)	171 (5.3)
Apostates	26 (5.2)	222 (6.9)
Disbelievers	254 (50.7)	1565 (48.3)

**Table 3:** Proportion of sample that maintained or changed their religious affiliation from 2009 to 2011 depending upon self-reported personal experience of the Christchurch earthquake.

Category	Result(%)	Result(%)
	Personally affected	Not personally affected
Believers	308 (38.5)	1140 (39.1)
Converts	59 (7.4)	153 (5.2)
Apostates	41 (5.1)	205 (7.0)
Disbelievers	393 (49.1)	1417 (48.6)

However the data on religious conversion in the Canterbury region present a strikingly different pattern, revealing a net increase of 3.4% in religious faith. A chi-square test indicated the difference in the rate change by region was significant (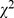
(3, N = 3740) = 12.39, p = .006). Thus, in the region where the church spires had fallen, faith soared.

We observed a similar trend among those who reported being personally affected by the Christchurch earthquakes (regardless of region). As shown in Figure 2., those who were personally affected by the Christchurch earthquakes were significantly more disposed to religion than were those who were not personally affected (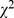
(3, N = 3716) = 8.51, p = .037). This result shows that the steady erosion of religious faith observed throughout New Zealand for the last forty years was dramatically reversed among those most directly impacted by the Christchurch earthquakes.

#### Distribution of effects for religious conversion

We examined whether changes in religious affiliation for participants who were affected by the earthquake, relative to those who were not affected by the earthquake, were broadly consistent across different demographic groups, or whether they were more pronounced for specific demographic groups, such as men versus women, people of different ages, or different ethnic groups. We failed to detect any reliable differences in rates of apostasy and conversion moderated by these demographic factors, although religious conversion was 1.1% higher for women during the 2009 to 2011 period overall (this did not differ in comparisons between the Canterbury region versus other regions, however). Religious conversion rates were also 1.3% higher amongst minority groups relative to the New Zealand European majority, although again, this did not differ by region. These findings indicate that religious conversion may be more pronounced for women and ethnic minority groups, but these demographic differences seem to be independent of the effect of the Christchurch earthquakes.

#### Effects of earthquake exposure on subjective health depending on religious faith

The religious conversion rate increased among those affected by the Christchurch earthquakes, but did having or acquiring a belief in God help? We focussed on subjective health evaluations, a key indicator of psychological well-being. To evaluate both superior and selective buffering effects we conducted a series of mixed-factor 2×2 ANOVA models comparing mean levels of subjective health evaluation in 2009 and again in 2011 (the repeated measures component) among those who were exposed or not exposed to the Christchurch earthquakes (the between persons component). We conducted these 2×2 models separately for believers, disbelievers, converts, and apostates. A significant interaction in any one of our ANOVA models would indicate that subjective health evaluations changed significantly over time, and that the extent of this change depended on exposure to the Christchurch earthquakes and their aftermath.

**Figure 3:**
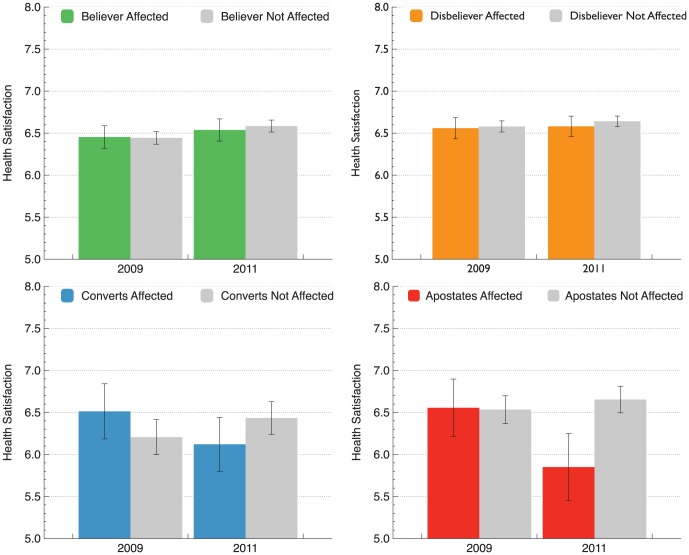
Changes in levels of subjective health evaluation from 2009 (pre-earthquake) to 2011(post-earthquake) for religious affiliates, non-religious affiliates, religious converts and religious apostates who reporting being personally affected versus not personally affected by the Christchurch earthquakes. Error bars represent the standard error of the mean.

The analysis focussing on self reports did not find a significant interaction between subjective health evaluations in 2009 and 2011 for religious believers (F(1,1428) = .17, p = .766, partial 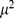
<.001). Nor were significant interactions found for converts (F(1,208) = 3.74, p = .054, partial 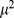 = .018). Nonsignificant results also emerged from the analysis of disbelievers (F(1,1795) = .11, p = .741, partial 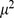
<.001). Overall these results did not support the Superior Buffering Hypothesis. However we found that among apostates there was a significant interaction between repeated assessments of subjective health and reports of being affected by the earthquakes (F(1,243) = 4.67, p = .032, partial 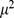 = .019). This interaction is represented in the lower right panel of Figure 3. As shown, the interaction between apostasy and the impact of the natural disasters occurred because subjective health evaluations decreased from 2009 to 2011 among the earthquake-affected apostates, but remained stable among all other groups, including apostates who did not reside in Christchurch. Consistent with selective buffering, a loss of faith in the wake of the Christchurch earthquakes was associated with reduced subjective health.

Our analysis applying the same method (ANOVA models testing) to regional data gave a similar result. The interactions were non-significant for believers (F(1,1439) = .02, p = .897, partial 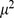
<.001), converts (F(1,210) = 2.08, p = .151, partial 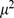 = .010), and disbelievers (F(1,1804) = .39, p = .533, partial 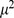
<.001). These findings suggest that religious faith did not offer any superior buffering advantages for subjective health among those who lived in close proximity to the natural disaster. By contrast, the regional analysis was again consistent with selective buffering. There was a significant interaction between repeated assessments of subjective health and region among apostates (F(1,245) = 6.21, p = .013, partial 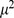 = .025). Apostates elsewhere did not exhibit similar declines in health. Consistent with a loss of buffering, it was only in the region of the Christchurch earthquakes that apostasy was associated with reduced global health evaluations.

## Discussion

### Religious comfort after a natural disaster

The Comfort Model of Religion has been the subject of longstanding philosophical debates. Several studies have shown that economic crises are associated with increases in religious practices [67]; other studies have shown that enhanced social and economic security are associated with declines in religious commitment [55], [58], [60]. Our study answers the call for “naturalistic methods” in the study of religion [70]. Using a longitudinal national sample, with time pre/post the earthquake as the within subjects variable and earthquake exposure as the between subjects variable, we found that religion became more appealing among those exposed to the Christchurch earthquakes, relative to those who were not exposed. This finding offers the first evidence from a large population that religious conversion increases following a natural disaster.

Despite the interest of these findings, we offer a few words of caution about their interpretation. First, conversion is likely to be a complicated process [71]. It has been observed that religious thinking and behaviours are variable both within and across populations [72], [73]. Following the earthquakes, not everyone turned to God and some turned away: the dramatic increase in conversion was a net effect.

Relatedly, “comfort,” as we have used this term, denotes a revealed preference for religion as quantified by conversion rates. The emotional mechanisms of religious commitment are complex [74]. Studies have shown that religious practices occasionally recruit strictly discomforting emotions, such as tragic recollections of the suffering of others, or suggestions that God suffers with the afflicted [75], [76]. Such religious grief might proportion natural grief. The mechanisms that underpin religion's appeal at times of exceptional collective hardship are matters for future investigations.

Keeping these provisos in mind, it is remarkable that there was a significant overall increase in religious faith among the earthquake-affected. Consider: a third of the buildings in Christchurch were destroyed, including the Catholic and Anglican Cathedrals. Homes and businesses were lost. Many hundreds were badly injured. One hundred and eighty-five people lost their lives. Philosophers have plausibly argued that natural disasters such as the Christchurch earthquake are rationally incompatible with the existence of an all-powerful, all-loving God, because natural disasters cause pointless suffering to innocents, which a compassionate power would never permit [77]–[79]. Though faith eroded elsewhere in New Zealand, there was a significant upturn in religious faith among those who experienced the misery of New Zealand's most lethal natural disaster in eighty years.

### Superior and selective religious buffering after a natural disaster

Other studies have found that in the aftermath of natural disasters, religious faith helps with post-traumatic recovery [80], increases positive emotions [81], and increases social connection [82]. Other research paints a more complex picture, suggesting that religious coping offers no special advantages over non-religious coping [57], and that recollected declines in the importance of religious belief are associated with poorer psychological well-being [69]. Longitudinal NZAVS survey data taken before and after the Christchurch earthquakes enabled us to examine change in faith and subjective well-being in the wake of a natural disaster. We found that merely possessing religious faith did not offer superior buffering advantages for subjective health, yet those who lost their faith and were affected by the Christchurch earthquakes experienced significant declines in subjective health. Unaffected apostates, however, did not experience similar declines. The combination of losing religious faith in the wake of a natural disaster differs from the combination of having or acquiring faith.

How was the loss of faith related to the earthquake? Recall that Seirmarco and colleauges observed declines in the reported importance of religious beliefs several years after September 11 attacks were associated with reported severity of personal loss from the tragedy [69]. In the aftermath of a horrific and deadly earthquake, it is plausible that those religious people who experienced the greatest losses to subjective well-being were overwhelmingly challenged in their faith, from excessive existential concerns and incongruities. It is also possible that erosions of faith and erosions of subjective health mutually elaborated each other through reciprocal processes. Finally, it is theoretically possible that the religious conversion group affected by the earthquake was over-represented with people who experienced significant hardships, yet who, by faith, were able to maintain subjective well-being – thus experiencing superior buffering. We could not experimentally manipulate religious affiliation and levels of earthquake hardship (clearly). Our data do not reveal why people converted. Yet we can infer that there were two parallel processes that coincided only in the Canterbury region and only among those who reported being affected by the earthquakes: loss of faith and decreases in subjective health. Respondents in other categories of religious affiliation remained constant in their subjective well-being, whether or not they were affected by the earthquakes.

Setting aside causal ambiguities, it is important to remember that it does not take an earthquake to lose faith. Indeed, those who experienced the Christchurch earthquakes were, on the whole, more likely to identify as religious. That there was some apostasy in Christchurch, moreover, accords with a longstanding trend to apostasy in New Zealand [52]. Our discovery that Christchurch apostates experienced declines in perceived health suggests that selective buffering is possible. The loss of religious faith might be harmful even if religious faith per se does not provide superior buffering. Before investigating the processes by which faith affects well-being in the wake of a devastating natural disaster, researchers should distinguish between the advantages of having faith and the disadvantages of losing faith.

It is important to recognise that our study leaves many questions unresolved. The association that we observed between loss of faith and diminished subjective well-being might be limited to New Zealand, or to similarly secular societies. Furthermore, the relationship between subjective well-being and biological health remains unknown. Perhaps appearances of stable health are linked to optimistic illusions [83], to cognitive dissonance [84], [85], or to some other tendency for “misbelief” [86]. Notably, we found that longstanding disbelievers maintained stable subjective health evaluations after the Christchurch earthquakes. Should positive illusions hold the key to understanding subjective health maintenance, disbelievers, too, might be prone to them [87]. Unfortunately, little at present is known about the processes that support a sense of personal well-being following a terrible natural disaster. Yet a clearer understanding of these processes is urgently needed if policy makers are to develop sensibly strategies for helping people to recover.

#### Importance of findings

Though many questions remain, we believe that our findings hold methodological, theoretical, and practical importance. Methodologically, our study reveals the power of within-country longitudinal designs to cast new light on fundamental questions of basic human concern. Within-country designs are complementary to transnational designs such as the World Values Survey, however within-country designs also offer distinctive advantages for addressing certain questions. Without access to regional data and personal reports, we simply could not have compared those who were affected by the earthquake with those who were not, and therefore, could not have evaluated the effects of the Christchurch earthquakes on religious faith and subjective health. Were we restricted to national averages, the signals of stability and change would have been obscured. We urge researchers in other countries to embark on national longitudinal projects, to better understand why core beliefs and values persist and change over time, and how their dynamics affect human flourishing.

At a theoretical level, our study contributes to the old philosophical question of whether people seek religious faith at times of crisis (they do). Moreover, longitudinal data enabled us to more precisely investigate whether religion actually helps the faithful. The association between apostasy and diminished personal health suggests the need to distinguish between the benefits of having a religious faith and the costs of losing it. Secularisation has been associated with increases to security and income. In highly secular, affluent, and peaceful democracies such as New Zealand, we might expect a range of philosophical and institutional supports to protect individuals at times of crisis. Whereas religious converts might benefit from ready-made theologies and clearly identifiable communities of support, secular communities appear to be more diffuse, and less clearly associated with normative systems. Yet benefits from secular buffers might therefore be harder to access. Comparative longitudinal data afforded by the NZAVS revealed that it was a combination of the natural disaster and a loss of faith that was responsible for Christchurch apostates' declining sense of personal health. We hope the distinction between superior and selective buffering proves helpful to researchers investigating the costs and benefits of religious faith.

Finally, our study is relevant to disaster relief. Sensible policies for addressing human misery rely on understanding the cultural and psychological resources available to those who need help. Though many questions remain unanswered, our results suggest that conversion to religious faith in the aftermath of a natural disaster is unlikely to improve subjective health, however de-conversion is associated with declines in subjective health. Whether religious or secular, those who want to foster healing in the aftermath of a devastating earthquake should consider joining arms in rebuilding the broken churches, for those who have faith.

## Methods

### Participants sampling procedure

The NZAVS-2009 questionnaire was posted to 40,500 participants from the 2009 NZ electoral roll. The publicly available version of the roll contained 2,986,546 registered voters. This represented all citizens over 18 years of age who were eligible to vote regardless of whether they chose to vote, barring people who had their contact details removed due to specific case-by-case concerns about privacy. In sum, roughly 1.36% of all people registered to vote in New Zealand were contacted and invited to participate. The NZAVS-2009 sampled a total of 6,518 participants. The overall response rate (adjusting for address accuracy of the electoral roll and including anonymous responses) was 16.6%. These participants were contacted by postal mail and invited to complete a follow-up questionnaire one year later. The NZAVS-2011 contained responses from 3,914 of the same participants, a retention rate of 60.04% over the two-year period from 2009 to 2011. We limited our analyses to the 3,745 participants who completed the questions regarding their religious affiliation in both 2009 and 2011. The sample analysed here consisted of 2,305 women and 1,440 men with a mean age of 50.14 years in 2009 (SD = 14.94). The majority of participants (78.8%, n = 2871) were NZ European/Pakeha. There were 14.2% (n = 532) identifying as Maori; 3.1% (n = 115) who identified with a Pacific Nation; 3.3% (n = 124) who identified as Asian; and 2.8% (n = 103) who identified as “other”. The education of participants reported in 2009 were as follows: 738 (19.7%) had no formal qualification, 1066 (28.5%) had a secondary school qualification, 606 (16.2%) had a tertiary diploma or trade certificate, 932 (24.9%) had an undergraduate university degree and 403 (10.8%) had a postgraduate qualification.

### Changes in the sample over time

Note that our sample was large (6,518 participants in 2009, with retention of 3,914 participants in 2011). However, the initial response rate at Time 1 was fairly low (16.6%) and the retention rate from 2009 to 2011 was 60%. The low initial response rate was most likely because the study aimed to recruit people for a planned 20-year longitudinal investigation. The 2009 sample was reasonably representative of differences in the proportion of ethnic groups according to 2006 census figures. However, Pacific and Asian respondents were underrepresented in the 2011 wave: people who identified with these groups were slightly more likely to drop out of the sample. The NZAVS also oversampled women relative to men; however, as we noted earlier, differential changes across regions in religious affiliation were consistent when examining men and women separately. These caveats should nevertheless be kept in mind when generalising from our sample to the New Zealand population.

### Religious affiliation and identification

We focussed on changes in religious affiliation depending on whether people were affected by the massive Christchurch earthquakes occurring in September 2010 and February 2011. Specifically, we examined change in religious affiliation both across regions and depending upon personal reports of whether participants were affected by the earthquakes. Our primary analyses focused on change in yes/no responses to the question “Do you identify with a religion and/or spiritual group?” asked in both late 2009 (before the earthquakes) and again to the same participants in late 2011. We thus identified four possible categories: Believers, Converts, Apostates, Disbelievers (see Figure 2). In both 2009 and 2011, we assessed religious affiliation by asking “Do you identify with a religion and/or spiritual group?” Participants who answered “yes?” were directed to an open-ended field in which they could specify their religious denomination or group membership. Religious denominations were coded using the statistical standard developed by Hoverd (2010) and Hoverd and Sibley (2012) [53], [88]. The religious sample was primarily Christian (93.5% n = 1469). The sample of religious groups (reported at Time 1) consisted of the following: Anglican (n = 303), Apostolic (n = 4), Assemblies of God (n = 5), Baha'i (n = 4), Baptist (n = 36), Born Again (n = 4), Brethren (n = 8), Buddhist (n = 20), Catholic (n = 376), Christian Other (n = 13), Christian NFD (n = 419), Church of Christ (n = 2), Congregationalist (n = 3), Evan/BA/Fund (n = 9), Freemason (n = 2), Hindu (n = 18), Jehovah's Witness (n = 14), Jewish (n = 2), Lutheran (n = 6), Maori Other Religion (n = 4), Methodist (n = 37), Mormon (n = 28), Muslim (n = 4), New Age (n = 1), New Life (n = 5), Orthodox (n = 4), Other Religion (n = 19), Pagan (n = 6), Pentecostal (n = 18) Presbyterian (n = 125), Quaker (n = 1), Ratana (n = 17), Reformed (n = 3), Ringatu (n = 1), Salvation Army (n = 14), Seventh Day Adventist (n = 12), Sikh (n = 2), Spiritual (n = 17), Union (n = 2) and Wiccan (n = 3).

### Experience of the Christchurch earthquakes

We measured exposure to the Christchurch earthquakes using two independent methods: (a) by identifying the region where participants were living in 2009 before the earthquakes; (b) by asking participants in the 2011 questionnaire to indicate whether or not they had been affected by the earthquakes. To measure self-reported earthquake exposure, the 2011 questionnaire asked, “were you personally affected by the Christchurch earthquakes?” allowing a yes/no response. We divided participant regions into two broad categories, those living in the Canterbury region in 2009 (Note: Christchurch is the central city in the Canterbury region). Of the longitudinal sample that we analysed (3,745) a total of 501 (13.4%) respondents lived in the Canterbury region. This proportion is consistent with 2006 census figures indicating that 12.9% of the population lived in this region prior to the earthquake. In total, 801 (21.4%) individuals in the sample reported being personally affected by the Christchurch earthquake. Notably, our two measures of potential earthquake exposure overlapped: 370 respondents both lived in the Canterbury region and reporting being personally affected by the earthquake. These measures potentially index different aspects of earthquake exposure, however, as one relates to region and the other taps broader experiences that relate to being personally affected regardless of residential location. We thus report parallel results for all analyses using both of these indicators. Importantly, we find nearly identical results from both the affectedness report analysis and from the regional analysis.

### Subjective health evaluation

We indexed subjective evaluations of health by asking participants to rate their satisfaction with their level of personal health on a scale from 0 (completely dissatisfied) to 10 (completely satisfied). This item was drawn from the broader scale indexing different aspect of personal wellbeing developed by Cummings (2003) [89], and had been used in New Zealand with various sub-populations (see [90], [91]).

### Ethics statement

The data reported in this study were collected as part of a larger longitudinal research project, The New Zealand Attitudes and Values Study. The first phases of this longitudinal study were approved by The University of Auckland Human Participants Ethics Committee on 09-September-2009 for 3 years, reference number: 2009/336. Ethics approval for the study was re-approved by the University of Auckland Human Participants Ethics Committee on 17-February-2012 until 09-September-2015. Reference number: 6171.
